# Supplementary figures and images for: Helper NLR immune protein NRC3 evolved to evade inhibition by a cyst nematode virulence effector
Source: PLoS Genet. 2025 Apr 9;21(4):e1011653. doi: 10.1371/journal.pgen.1011653 (PMC11981194; doi:10.1371/journal.pgen.1011653)

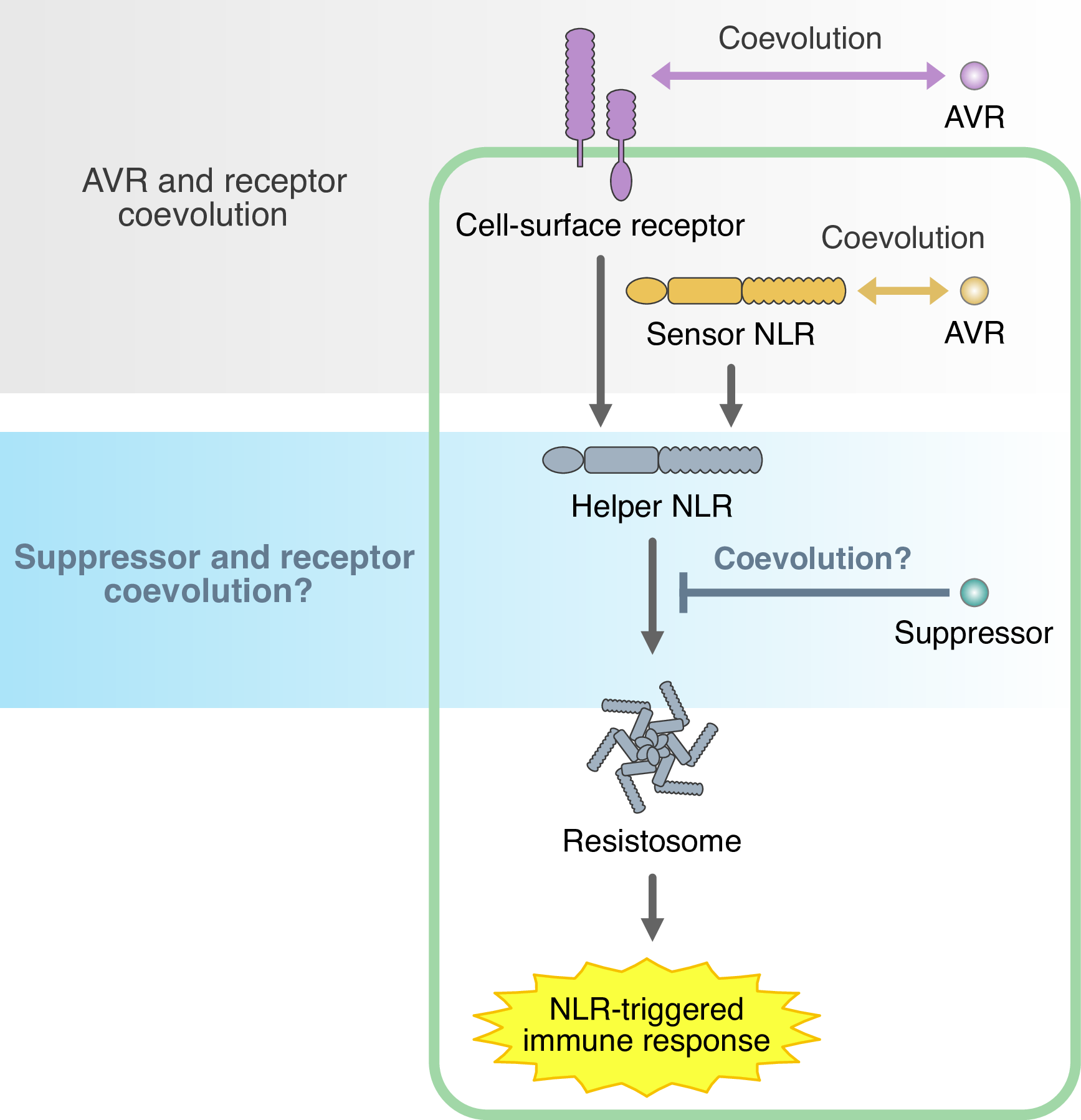

Supplement: S1 Fig — To date, most studies have focused on coevolution between R (immune receptors or R genes) and AVR effectors, of which there are numerous examples. On the other hand, coevolution between pathogen effectors with immunosuppression activities (suppressors) and their receptor targets are less understood. (TIF) [file pgen.1011653.s001.tif]

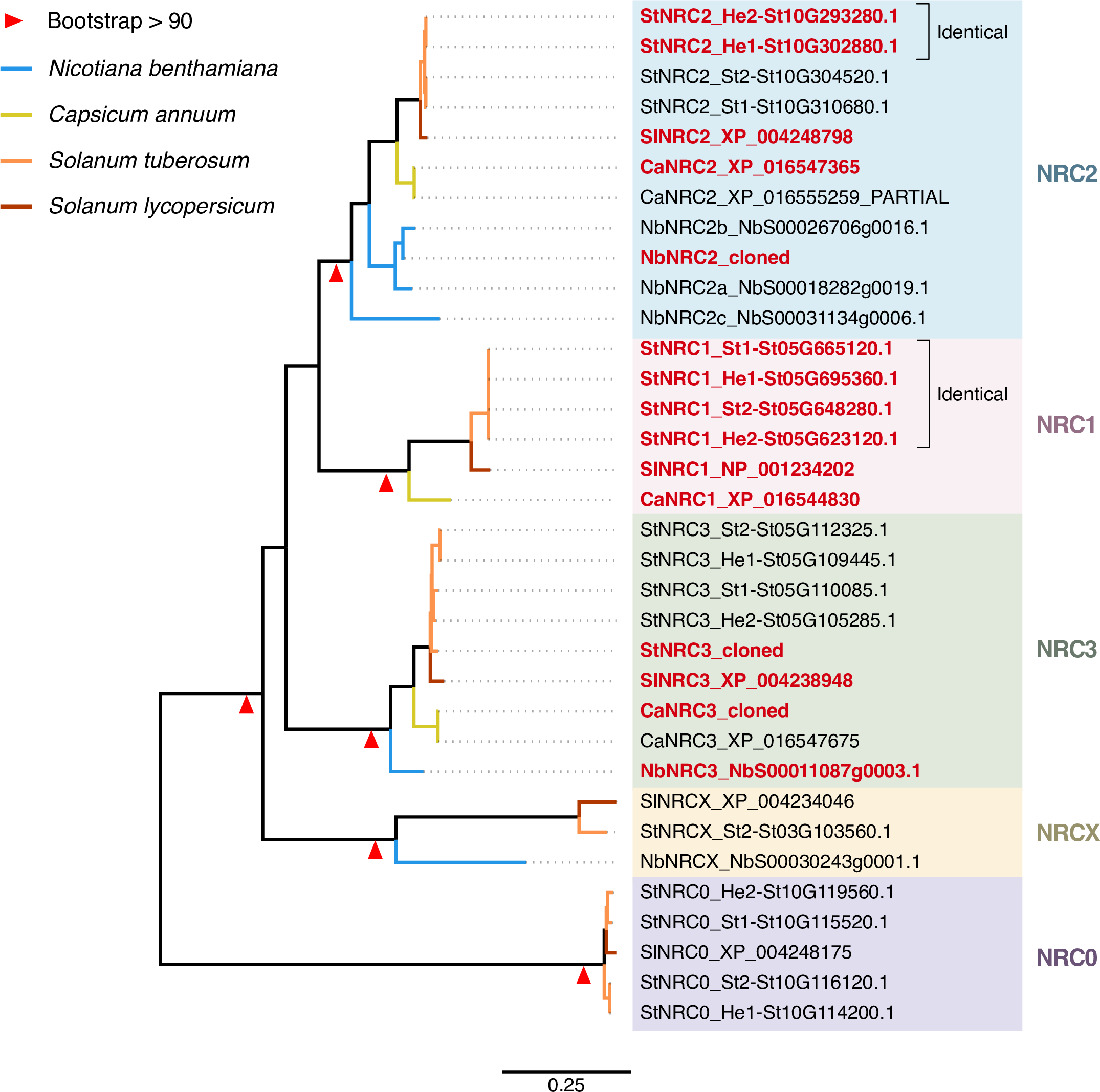

Supplement: S2 Fig — We used the NRC sequences of the NRC0, NRC1, NRC2, NRC3 and NRCX clades from four different Solanaceae species (Nicotiana benthamiana, Capsicum annuum, Solanum tuberosum and Solanum lycopersicum) and created a phylogenetic tree. When a cloned sequence is not identical to any sequences in the dataset, we added them to the dataset. Each cloned NRC sequence is highlighted in red. When a cloned sequence is identical to multiple NRC sequences in the tree, they are indicated in the figure. The phylogenetic tree is reconstructed by the maximum likelihood method using IQ-TREE with 1,000 bootstrap replicates. (TIF) [file pgen.1011653.s002.tif]

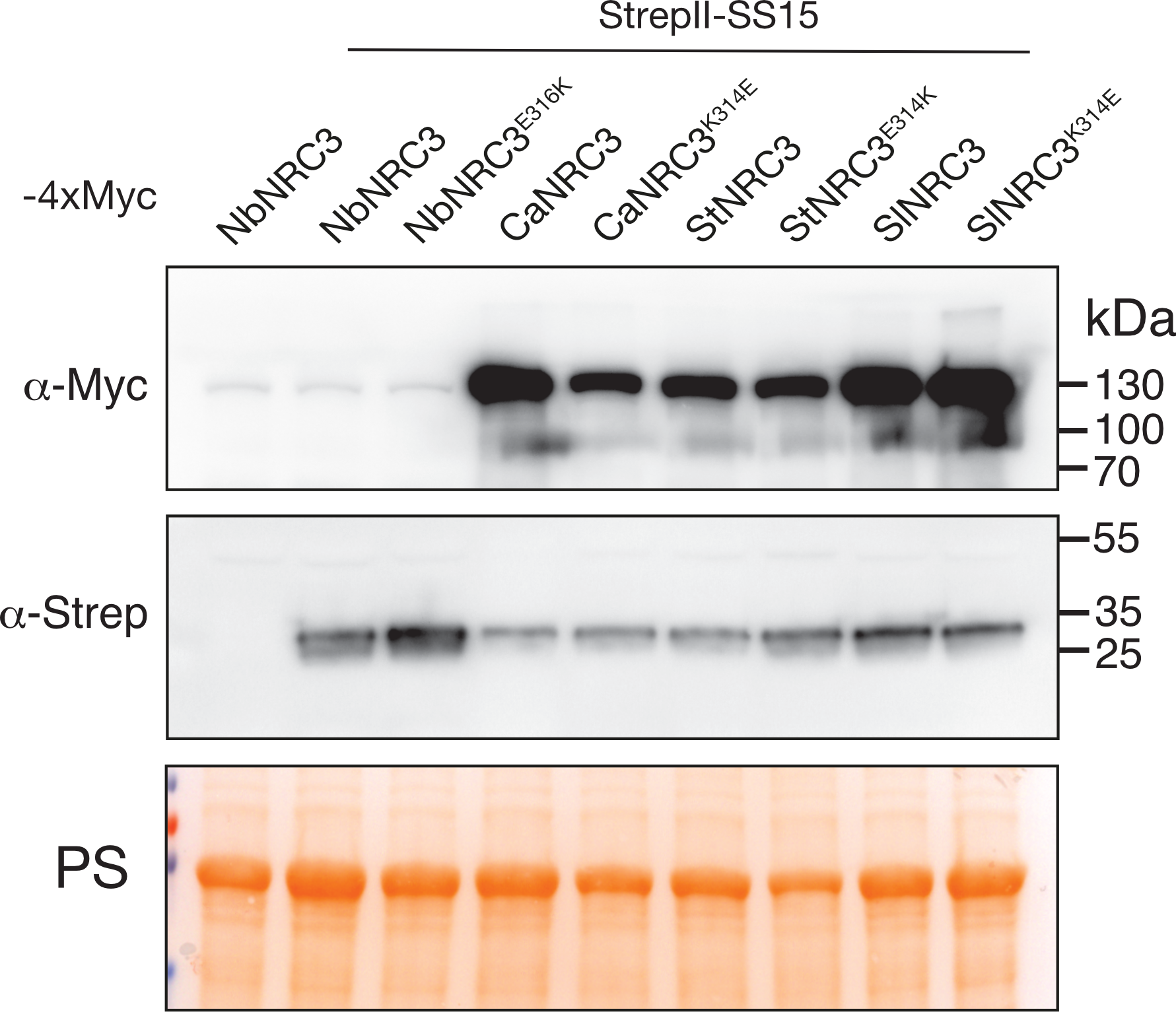

Supplement: S3 Fig — C-terminally 4xMyc-tagged NRC3s and single-point mutants were expressed with N-terminally StrepII-tagged SS15 in the leaves of N. benthamiana nrc2/3/4 KO plants. NbNRC3 expressed with EV was used as a negative control for the anti-Strep blot. Rubisco loading control was carried out using Ponceau staining (PS). (TIF) [file pgen.1011653.s003.tif]

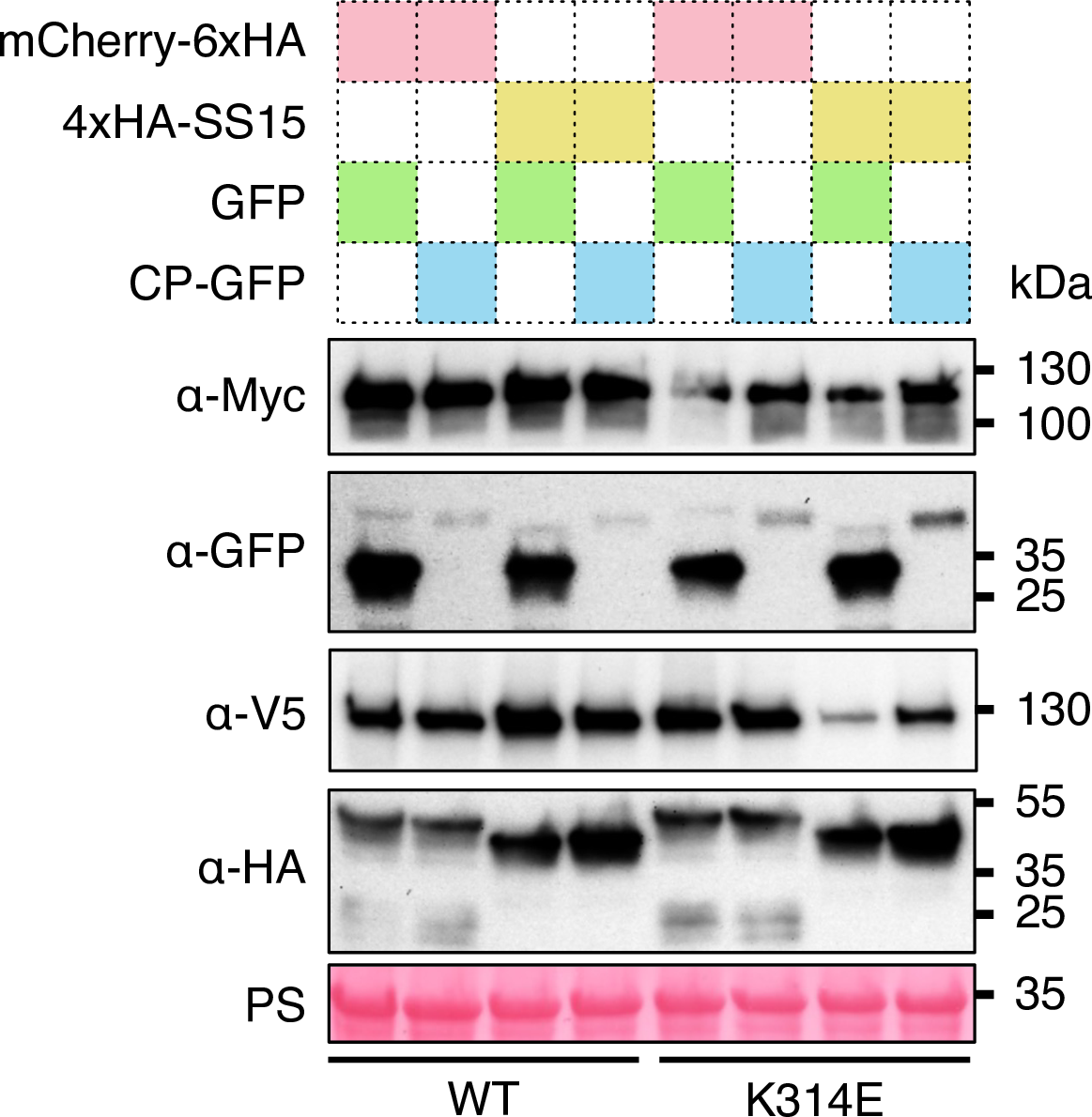

Supplement: S4 Fig — SDS-PAGE assay was conducted for CaNRC3EEE and its single-point mutant at position matching NbNRC3 residue 316. CaNRC3EEE represents the CaNRC3 mutant of the N-terminal MADA motif. C-terminally 4xMyc-tagged CaNRC3EEE mutants were co-expressed with C-terminally V5-tagged Rx and either free GFP or C-terminally GFP-tagged PVX CP in the leaves of N. benthamiana nrc2/3/4 KO plants. These effector-sensor-helper combinations were co-expressed either with mCherry-6xHA fusion protein or N-terminally 4xHA-tagged SS15. Rubisco loading control was carried out using Ponceau staining (PS). (TIF) [file pgen.1011653.s004.tif]

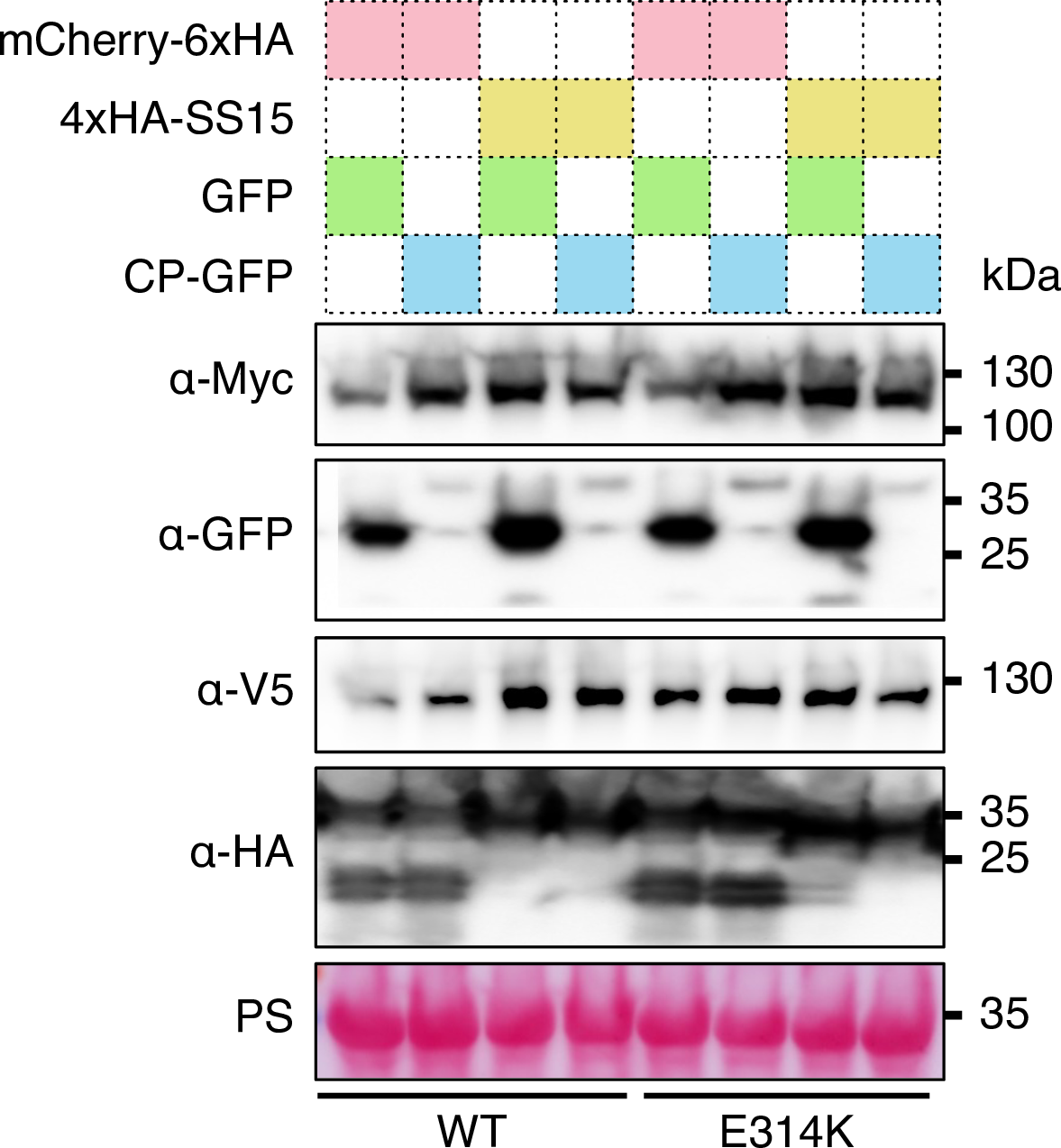

Supplement: S5 Fig — SDS-PAGE assay was conducted for StNRC3EEE and its single-point mutant at position matching NbNRC3 residue 316. StNRC3EEE represents the StNRC3 mutant of the N-terminal MADA motif. C-terminally 4xMyc-tagged StNRC3EEE mutants were co-expressed with C-terminally V5-tagged Rx and either free GFP or C-terminally GFP-tagged PVX CP in the leaves of N. benthamiana nrc2/3/4 KO plants. These effector-sensor-helper combinations were co-expressed either with mCherry-6xHA fusion protein or N-terminally 4xHA-tagged SS15. Rubisco loading control was carried out using Ponceau staining (PS). (TIF) [file pgen.1011653.s005.tif]

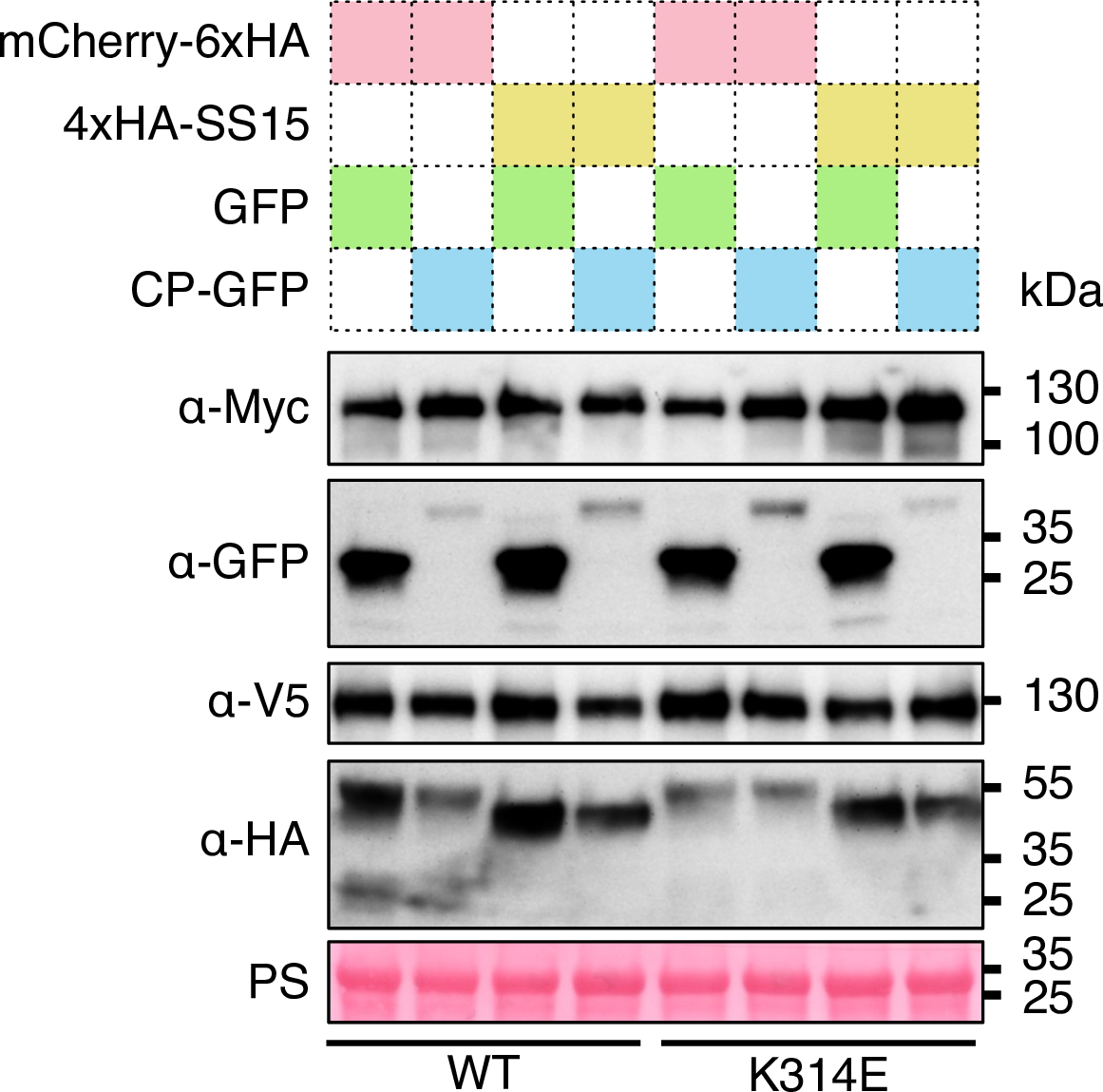

Supplement: S6 Fig — SDS-PAGE assay was conducted for SlNRC3EEE and its single-point mutant at position matching NbNRC3 residue 316. SlNRC3EEE represents the SlNRC3 mutant of the N-terminal MADA motif. C-terminally 4xMyc-tagged SlNRC3EEE mutants were co-expressed with C-terminally V5-tagged Rx and either free GFP or C-terminally GFP-tagged PVX CP in the leaves of N. benthamiana nrc2/3/4 KO plants. These effector-sensor-helper combinations were co-expressed either with mCherry-6xHA fusion protein or N-terminally 4xHA-tagged SS15. Rubisco loading control was carried out using Ponceau staining (PS). (TIF) [file pgen.1011653.s006.tif]

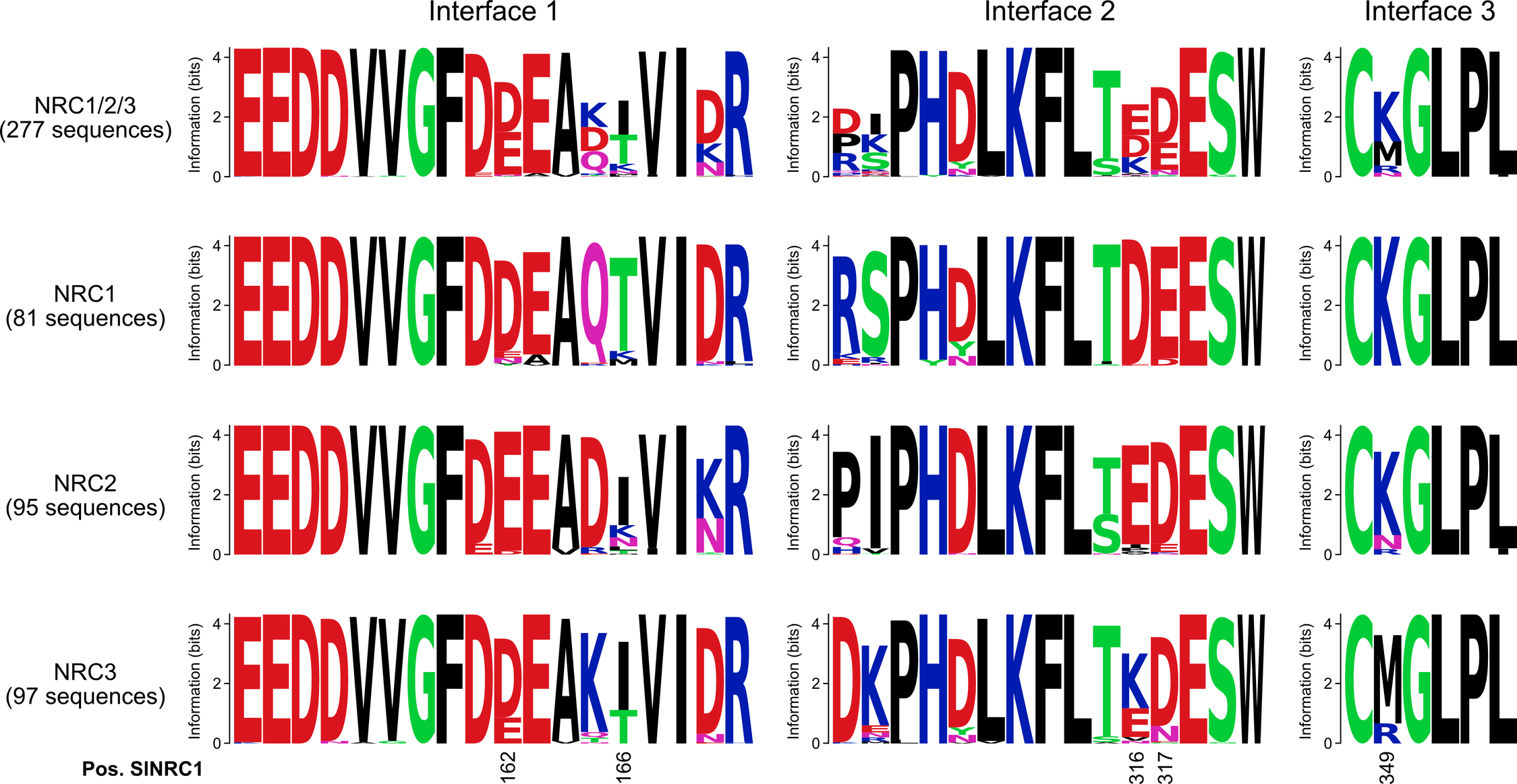

Supplement: S7 Fig — The consensus sequence patterns of each NRC clade and the NRC1/2/3 clade were generated using logomaker [93]. We used the NRC sequences and alignment from Fig 5A. The NRC1/2/3 clade was distinguished from the NRCX clade, where four sequences were not clearly assigned to either the NRC1, NRC2, or NRC3 clade (Fig 5A). (TIF) [file pgen.1011653.s007.tif]

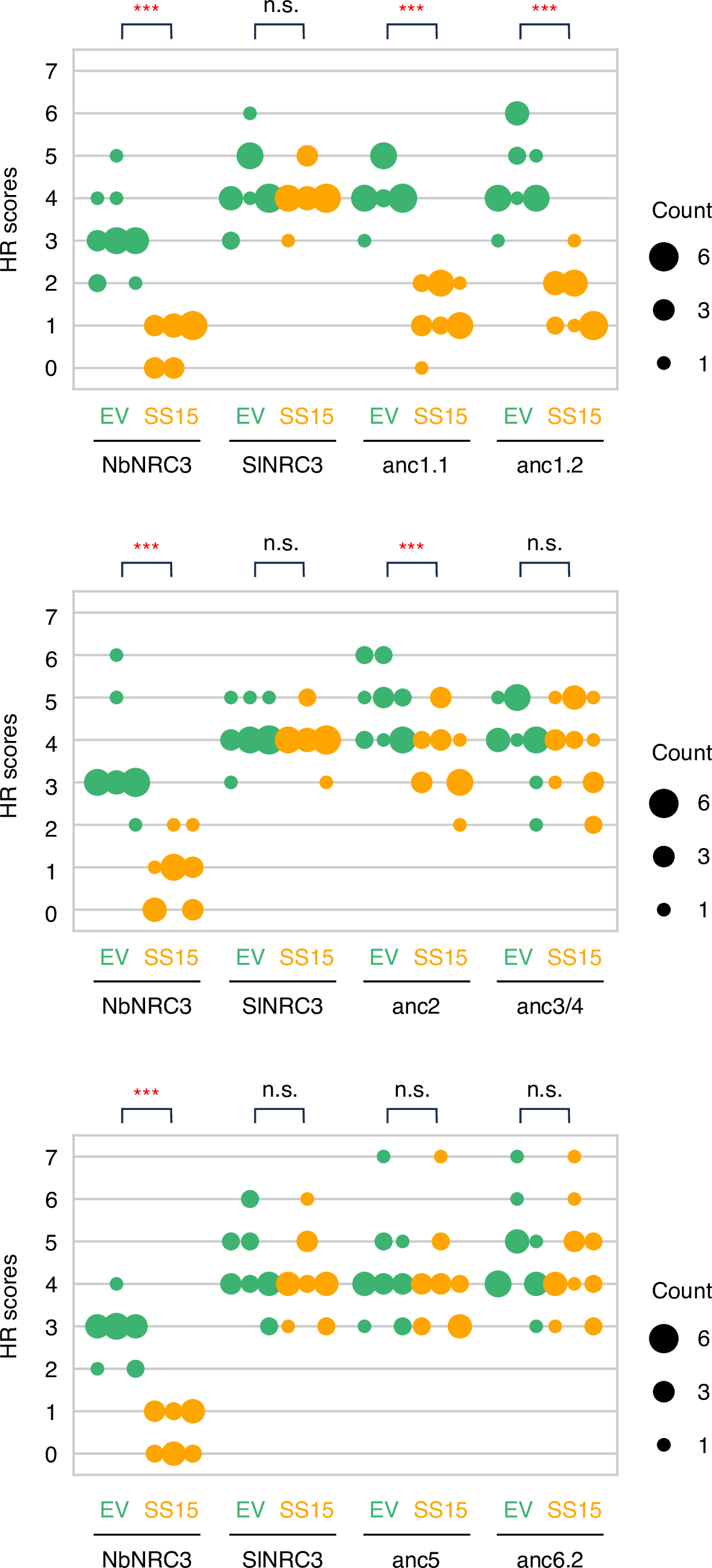

Supplement: S8 Fig — We used a two-sided permutation test with 10,000 replicates. Statistically significant differences are indicated (***: p < 0.001; n.s.: not significant). Each column represents an independent experiment. The data underlying this figure can be found in S1 Data. (TIF) [file pgen.1011653.s008.tif]

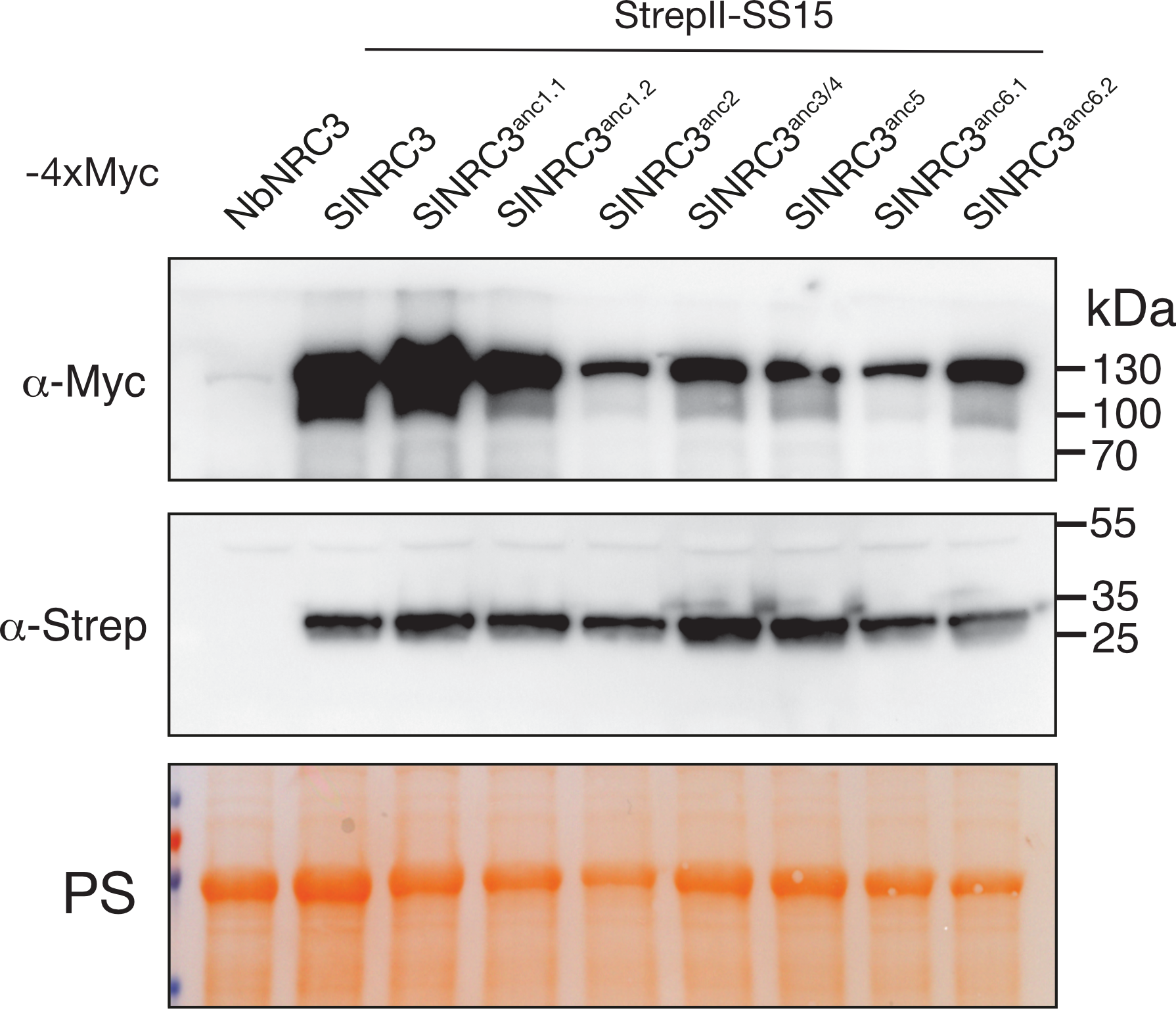

Supplement: S9 Fig — C-terminally 4xMyc-tagged ancestral NRC3 variants were expressed with N-terminally StrepII-tagged SS15 in the leaves of N. benthamiana nrc2/3/4 KO plants. The ancestral NRC3 variant for anc6.1 was identical to WT SlNRC3 at the five SS15 binding interface residues. NbNRC3 expressed with EV was used as a negative control for the anti-Strep blot. Rubisco loading control was carried out using Ponceau staining (PS). (TIF) [file pgen.1011653.s009.tif]

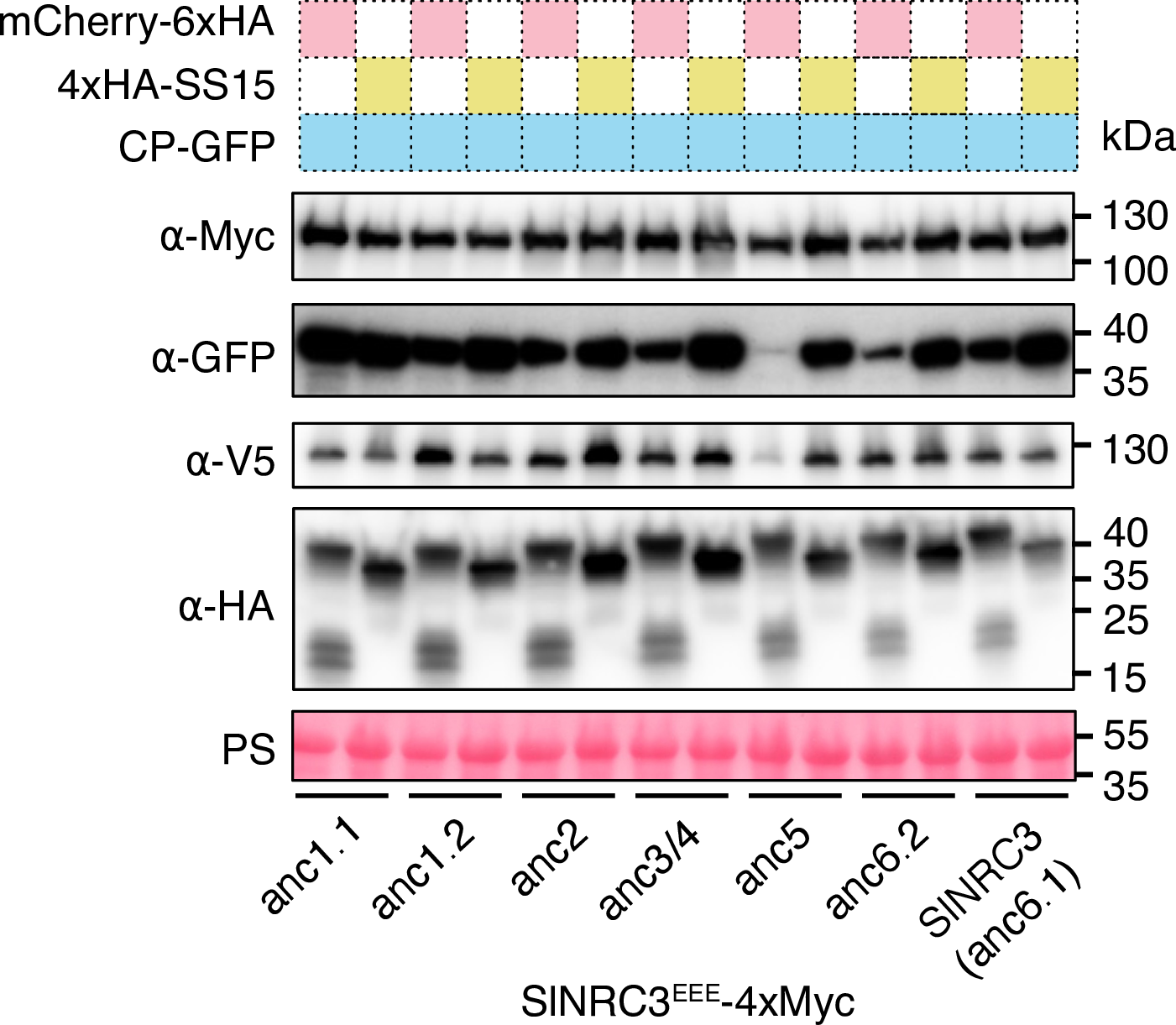

Supplement: S10 Fig — SDS-PAGE assay was conducted for ancestral NRC3EEE variants. NRC3EEE represents the NRC3 mutant of the N-terminal MADA motif. C-terminally 4xMyc-tagged ancestral NRC3EEE variants were co-expressed with C-terminally V5-tagged Rx and C-terminally GFP-tagged PVX CP in the leaves of N. benthamiana nrc2/3/4 KO plants. These effector-sensor-helper combinations were co-expressed either with mCherry-6xHA fusion protein or N-terminally 4xHA-tagged SS15. (TIF) [file pgen.1011653.s010.tif]

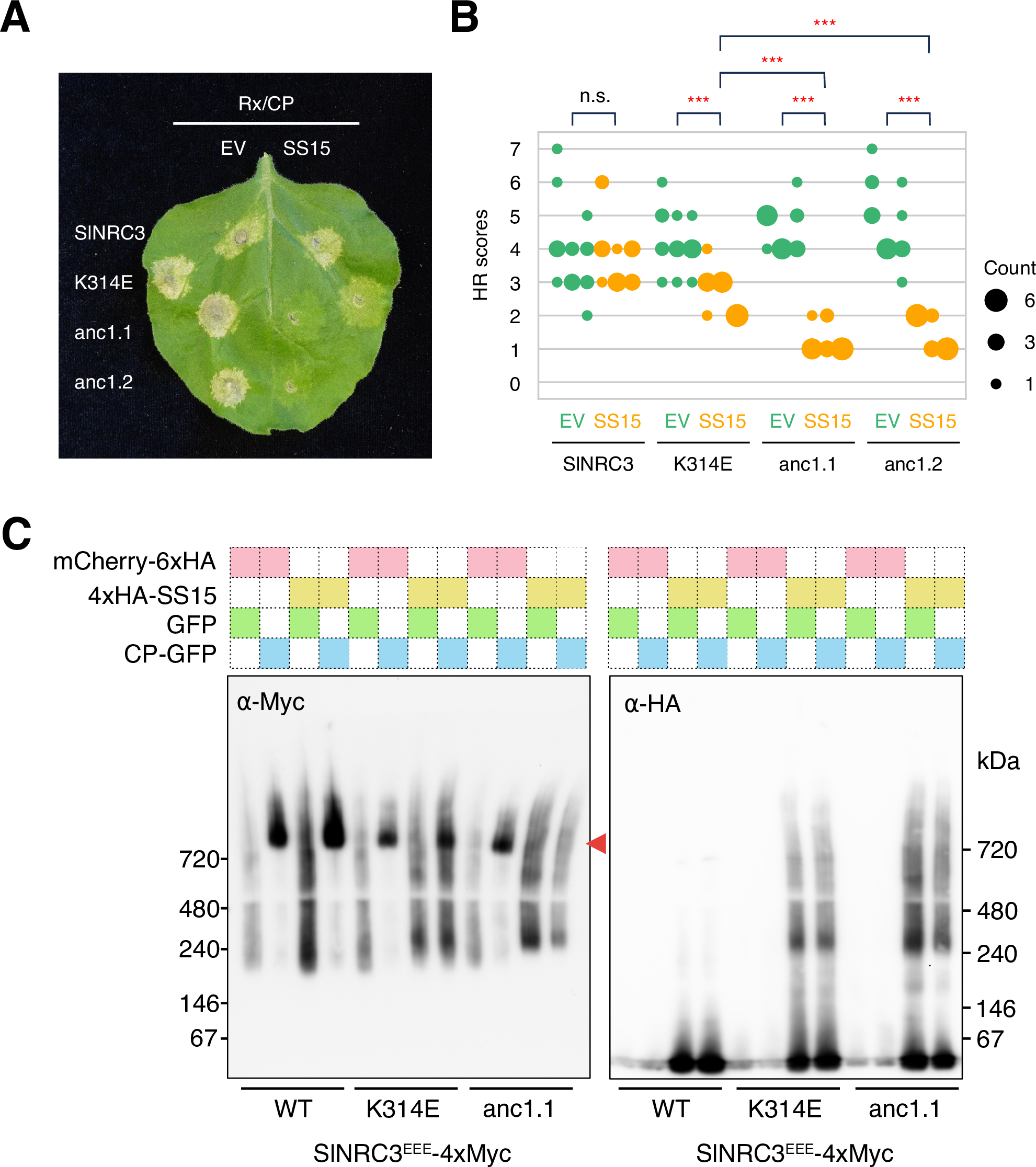

Supplement: S11 Fig — (A) Representative images of HR cell death assays showing the results after transient co-expression of either an empty vector (EV) or SS15 with Rx and PVX CP, along with either WT SlNRC3, SlNRC3K314E or the ancestral NRC3 variants (anc1.1 and anc1.2) in the leaves of N. benthamiana nrc2/3/4 KO plants. The anc1.1 and anc1.2 are the ancestral NRC3 variants tested in Fig 6. The leaves were photographed 5 days after infiltration. (B) Statistical analysis of S11A Fig using a two-sided permutation test with 10,000 replicates. Statistically significant differences are indicated (***: p < 0.001; n.s.: not significant). Each column represents an independent experiment. (C) BN-PAGE assays for WT SlNRC3, SlNRC3K314E and anc1.1. The SlNRC3EEEs, the N-terminal MADA motif mutants, were used in this BN-PAGE assay. C-terminally 4xMyc-tagged NRC3s were co-expressed with C-terminally V5-tagged Rx and C-terminally GFP-tagged PVX CP in the leaves of N. benthamiana nrc2/3/4 KO plants. These effector-sensor-helper combinations were co-expressed either with mCherry-6xHA fusion protein or N-terminally 4xHA-tagged SS15. A red arrowhead indicates resistosome bands. Corresponding SDS-PAGE blots are in S12 Fig. The data underlying S11B Fig can be found in S1 Data. (TIF) [file pgen.1011653.s011.tif]

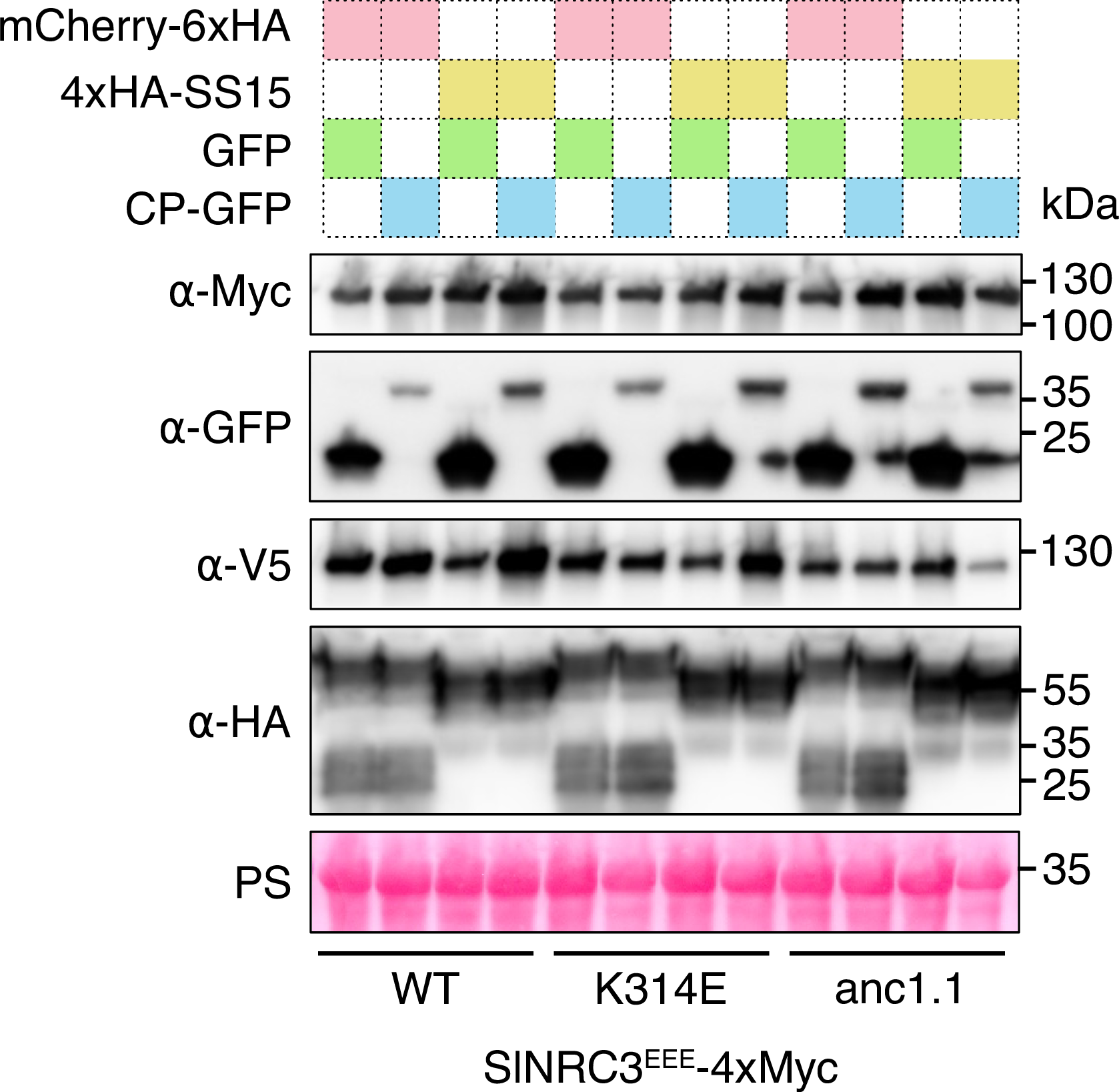

Supplement: S12 Fig — SDS-PAGE assays were conducted for WT SlNRC3, SlNRC3K314E and anc1.1. The SlNRC3EEEs, the N-terminal MADA motif mutants, were used in this SDS-PAGE assay. C-terminally 4xMyc-tagged NRC3 variants were co-expressed with C-terminally V5-tagged Rx and either free GFP or C-terminally GFP-tagged PVX CP in the leaves of N. benthamiana nrc2/3/4 KO plants. These effector-sensor-helper combinations were co-expressed either with mCherry-6xHA fusion protein or N-terminally 4xHA-tagged SS15. Rubisco loading control was carried out using Ponceau staining (PS). (TIF) [file pgen.1011653.s012.tif]

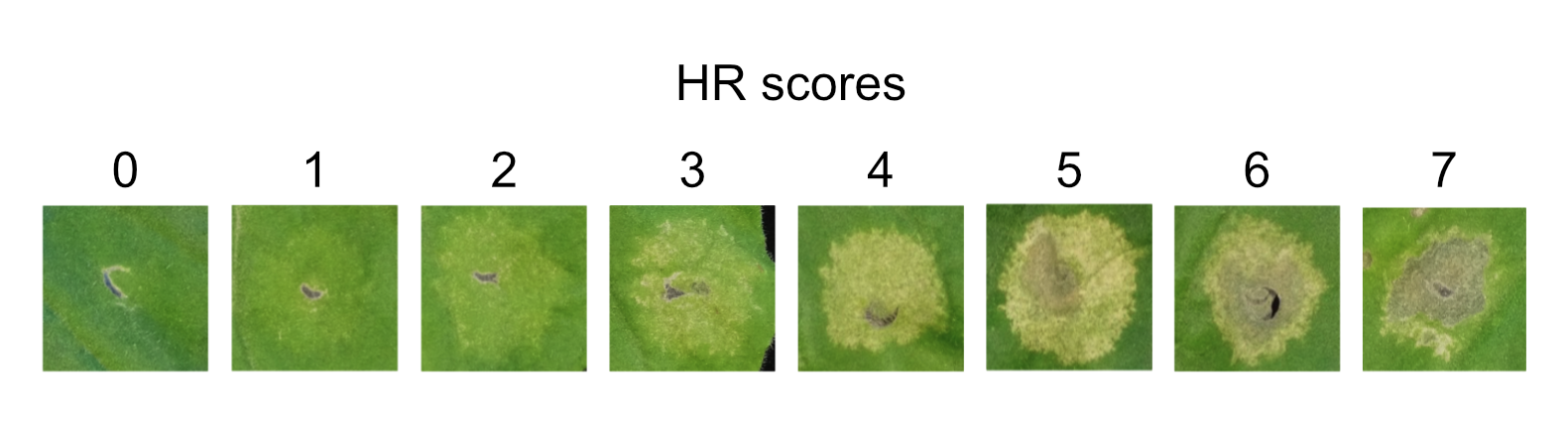

Supplement: S13 Fig — Cell death, HR, was photographed 5 days post infiltration and scored according to the 0 (no necrosis) to 7 (confluent necrosis) scale as described in previous studies [41,78]. (TIF) [file pgen.1011653.s013.tif]
