# Supplementary material for: Helper NLR immune protein NRC3 evolved to evade inhibition by a cyst nematode virulence effector
Source: PLoS Genet. 2025 Apr 9;21(4):e1011653. doi: 10.1371/journal.pgen.1011653 (PMC11981194; doi:10.1371/journal.pgen.1011653)
Supplement: Attachment — Submitted filename: PLOS Genetics Review.docx [file pgen.1011653.s017.docx]

The research addresses how parasitic nematodes suppress plant immune receptors by targeting NLR proteins, specifically focusing on SPRYSEC15 (SS15) as a suppressor. This focus is relevant to plant immunity. The manuscript is a follow-up of the previous study by Contreras et al 2023. In the previous manuscript, they examined the interaction of NLR and SS15 focusing on NRC2. The current study they concentrate on the NRC clade, focusing on NRC3.

The study shows how NRC1 and NRC3 proteins from various plant species exhibit different responses to SS15, with some variants being insensitive. They claim that this highlights evolutionary diversity within the NRC family in the Solanaceae species related to the interaction with SS15.

In previous studies, they examined the roles of positions D317 and E316 in NRC2. In this manuscript, they investigate how structural polymorphisms, particularly at positions E316 and D317 within the NB-ARC domain, are linked to the resistance of certain NRC proteins to SS15 suppression. The E316K substitution in NRC3 appears to be critical for modifying sensitivity across species, with lysine (K) at position 316 being associated with resistance to SS15.The authors show that polymorphism at NRC3 position 316 determines the ability of SS15 to suppress NCR3 function.

This provides a clear example of how a single amino acid can dictate protein-protein interactions. Throughout the evolution of NRC3, only two amino acids, K or E, have been present at this position, suggesting that this site was likely not under selective pressure to inhibit SS15 binding during evolution

I find this manuscript very interesting. Finding that position 316 is important to the function of the NRC protein can enhance our understanding of its function.

Weaknesses:

While the study focuses on the evolutionary dynamics of NRC proteins and their interaction with SS15, it doesn’t explore how these findings might influence crop breeding strategies to enhance disease resistance in agriculture.

The role of other residues beyond E316K is briefly touched upon (e.g., K314 in SlNRC3), but further exploration of these polymorphisms could strengthen the understanding of SS15 resistance. Their contribution to the insensitivity of tomato NRC3 could be elaborated.

The conclusions are based on transient expression assays, and it would be beneficial to discuss how these results might differ in stable transformation systems.

The manuscript is challenging to follow due to the excessive use of abbreviations. For example, what is the difference between NRC3 and NRC3^EEE^. The label in Figures 4, 6 S4-8, is confusing. Instead of specifying which antibody was used, it would be clearer to label the detected proteins directly.

Fig 3c-. Please elaborate on the experiment, the samples, the counts, and how you defined the scale of HR scores ("Cell death and suppression assays"- method section is unclear about the HR scores between 1-6).

Evolutionary trade-offs of E-K transition- is it associated with other aspects of plant fitness?
