# Supplementary material for: Helper NLR immune protein NRC3 evolved to evade inhibition by a cyst nematode virulence effector
Source: PLoS Genet. 2025 Apr 9;21(4):e1011653. doi: 10.1371/journal.pgen.1011653 (PMC11981194; doi:10.1371/journal.pgen.1011653)
Supplement: Attachment — Submitted filename: 24-01-14_point-by-point.docx [file pgen.1011653.s019.docx]

We have thoroughly revised the manuscript to address the reviewers' comments and have provided point-by-point responses to each concern. Major revisions include adding data on the protein accumulation levels of WT NRC3s and their mutants (Fig S3 and S9), as per Reviewer #1's suggestion. Additionally, we included logo plots showing sequence diversity at the interfaces against SS15 and the HR scale, as suggested by Reviewer #2 and #3, respectively.

**Reviewer #1:** In this study, while the cyst nematode virulence effector SPRYSEC15 (SS15) binds and inhibits oligomerisation of helper NLR proteins, the authors found some natural variants of NRC1 and NRC3 are insensitive to SS15 suppression. Further, they claimed that polymorphisms at NRC3 position 316 determine sensitivity to SS15 suppression by influencing NRC3-SS15 interaction. Additionally, ancestral sequence reconstruction revealed that NRC3 transitioned from an ancestral suppressed form to an insensitive one over 19 million years ago, suggesting the intricate and multilayered coevolutionary dynamics between NLRs and pathogen effectors. The concept of the story is intriguing; however, some important experiments are missing and should be supplemented.

Thank you for your valuable comments. In response, we have incorporated new data on the protein accumulation levels of NRC3s and their mutants (Fig S3 and S9).

1. The protein level of NRC3 and mutants in all HR cell death assays should be tested to exclude the instability or expression differences of mutants co-expression with SS15.

In the BN-PAGE assays, we have already confirmed the protein accumulation levels of NRC3^EEE^ mutants in the presence and absence of SS15 (Fig. S4–S6, S10), which addresses your concern. To further address your concern, we have added new data from western blots showing the protein accumulation levels of WT NRC3s and their mutants with SS15 (Fig. S3 and S9). Consistently, we did not observe any correlation between SS15 suppression levels and protein accumulation levels, suggesting that the suppression is not due to SS15-mediated destabilization of NRCs.

2. The authors claimed E316K substitution on NRC3 influence NRC3-SS15 binding, thereby influencing SS15 inhibition of NRC3 mediated HR cell death. However, the data of SS15 interaction with NRC3 or variants were missing.

BN-PAGE assays (Fig 4) demonstrate the effect of E316K on NRC3-SS15 interactions. In Fig 4, α-HA detection shows high molecular weight bands in CaNRC3^EEE/K314E^, StNRC3^EEE^, and SlNRC3^EEE/K314E^, which are inhibited by SS15. These bands are absent in CaNRC3^EEE^, StNRC3^EEE/E314K^, and SlNRC3^EEE^, which are insensitive to SS15. This pattern indicates that the E316K mutation alters SS15 binding. Additionally, Contreras et al. (2023, https://doi.org/10.1126/sciadv.adg3861) demonstrated that SS15 directly binds and inhibits NRCs, and mutations at the SS15 interface can affect this binding.

3. Since NRC3 is a core helper NLR required not only by sensor NLRs but also by cell surface receptors, the subcellular localization of NRC3 & mutants is critical to their function and should be examined.

Our study specifically focuses on NRC3-SS15 interactions rather than NRC3’s interactions with cell surface receptors or sensor NLRs.

4. In their study, the effects of single-point mutants (E316K in NbNRC3, K314E in CaNRC3, E314K in StNRC3) are remarkable in HR cell death assays and BN-PAGE assays (Fig2B, Fig4, Fig6B-C), suggesting position 316 (K or E) determines NRC3 oligomerisation and sensitivity to SS15 inhibition. Since Contreras et al. (2023) described the three contact interfaces in the SlNRC1NB-ARC complex. How could one amino acid residue plays such the dominant role?

The importance of positions 316 and 317 has been demonstrated in Contreras et al., 2023 (Fig. 3 and 4, https://doi.org/10.1126/sciadv.adg3861). Position 316 is located at the interface for SS15 and could serve as a hot-spot for protein-protein interactions. Therefore, the E316K substitution likely interferes with SS15 binding.

**Reviewer #2:** I previously reviewed the manuscript for a different publication and had an overall positive opinion, which holds true for this submission. I provide the same comments below, with edits after reviewing the present version.

The NRC sensor-helper network is a well characterized set of plant NLR receptors. Diverse sensor NRCs are related to, and genetically dependent on, a more conserved clade of helper NRCs, and the proteins make up an immunity module which has evolved many different pathogen recognition functions in Solanaceous plants. Recent landmark studies showed that a nematode efector SS15 and an oomycete effector can suppress immune responses through interaction with helper NRCs, and in the case of SS15 the interaction blocks the formation of the oligomeric helper NRC3 resistosome.

The present manuscript describes SS15 specificity against a broad range of helper NRC homologs (beyond the previously studied N.benthamiana paralogs). Naturally occuring SS15-evasive NRC3 homologs show HR insensitivity and BN-page oligomer resistance to SS15 perturbation. Through extensive phylogenomic analysis and functional testing of point mutants, the manuscript further shows that a specific NRC3 NBARC interface evolved the SS15-evasive mutation in the ancestor of Physalis / Capsicum / Solanum genera (E316K), but has undergone regressive evolution in potato StNRC3 (K316E) to again become senstive to SS15. Ancestrally reconstructed sequences at the interface are swapped into the SlNRC3 backbone to elegantly show a series of SS15 inhibition and evasion events over an inferred evolutionary timeline.

Overall the work is of high quality. Results are very clearly presented. The findings are impactful for several reasons. First, a broad survey of NBARC sequence variation and evolution motivates interest across all plant and animal NLRs at homologous HD1 subdomain interfaces, since the surface may serve as a target for other effectors to suppress oligomerization. Second, combining phylogenomic inference with structural models (empirical or predicted) is also an timely, powerful pipeline. The manuscript describes an excellent github repository "ancseq" to help other researchers use the pipeline. As usual for the research group, supplemental figures and supporting Zenodo datasets are easily accessible. I have 5 relatively minor comments.

We appreciate your positive comments and for taking the time to review our manuscript once again.

1) A limitation of the study is that only a single extant SS15 sequence is used from yellow potato cyst nematode, Globodera rostochiensis. The ancestral SS15 variants which may have formed interfaces with NRC3 ancestors are not explored.

This study focuses on the evolutionary trajectory of NRC3. Since it is uncertain whether ancestral SS15 could bind to ancestral NRC3, we have acknowledged this uncertainty in the discussion (lines 462–464).

The manuscript could add data on extant SS15 sequence diversity, and whether an ancestral SS15 was likely to have similar specificity against NRC3. At least two Globodera rostochiensis genomes are available at DOI: 10.1094/PHYTO-09-20-0403-A (raw reads could be searched by SRA blast), and related Globodera also have sequenced genomes <http://nemaplex.ucdavis.edu/Taxadata/G053.aspx>.

Thank you for your excellent question. The diversity of SS15 is indeed fascinating, and we are currently analyzing the sequence diversity of SS15 across several *Globodera* species, including *G. rostochiensis*, as part of a future study. However, this analysis goes beyond the scope and focus of the current paper and is therefore not addressed in this work.

2) The results imply that there is pressure on the key "toggling" residue 316 to revert to E even if the evasive K316 substitution exists. A discussion paragraph hypothesizes that K316 may confer a degree of autoactivity or autoimmunity, driving regression to E in the absence of the pathogen. If this is the case, would one predict that K316 variants are "trigger happy"? To test this, an experiment using lower levels of Rx/CP (lower OD of agroinfiltration) might be sufficient to activate similar levels of HR when K316 NRC3 variants are expresed, compared to E316. This experiment, or another demonstration of an advantage of E316, would help to explain the conflicting evolutionary pressures. An alternative hypothesis is that arms race dynamics with a specific electrostatic interaction with SS15 are driving reversion.

Thank you very much for your insightful comment. Some potato and tomato NRCs have been reported to exhibit mild autoactivity (see S3 Fig in Huang et al., 2024, https://doi.org/10.1371/journal.pgen.1011402 and Figure S4E in Sakai et al., 2024, https://doi.org/10.1093/plcell/koae154). However, these autoactivities have been observed in the transiently expression assays in *N. benthamiana*, and it remains unclear whether they occur in native plants. Since testing the "trigger happy" phenotype in native plants is challenging, we have decided not to pursue this analysis in the current study.

3) The reconstructed sequences of nodes 1-6 in Fig 5 were inferred from a larger alignment of 314 sequences of the NRC1/2/3/X clade (Fig. S3). Rather than presenting a schematic diagram of just 8 homologs, it would be better to move Fig. S3 to the main text to show the true number of NRC3 homologs used for inference.

Thank you for the helpful suggestion. In response, we have merged Fig. S3 into Fig. 5, as per your suggestion.

4) To visualize sequence conservation and diversity for NRC1/2/3 clades for the entire 314 sequence dataset, sequence logos for interfaces 1-3 could be added to Fig. 2. (Similar to Fig. 4B of Selvaraj et al 2023)

Thank you for your helpful suggestion. We've added the logo plots for the three interfaces as a new supplementary figure (S7 Fig).

5) Line 314: What is the overall sequence similarity of ancestrally reconstructed sequences to extant NRC3? An alignment figure of all ancestral sequences could be added as a supplemental figure.

The polymorphisms shown in Fig 5B represent all the variations on interfaces 1–3 detected by ancestral sequence reconstruction. As mentioned in the legend of Fig 5, the other residues on the interfaces are identical to SlNRC3.

**Reviewer #3:** The research addresses how parasitic nematodes suppress plant immune receptors by targeting NLR proteins, specifically focusing on SPRYSEC15 (SS15) as a suppressor. This focus is relevant to plant immunity. The manuscript is a follow-up of the previous study by Contreras et al 2023. In the previous manuscript, they examined the interaction of NLR and SS15 focusing on NRC2. The current study they concentrate on the NRC clade, focusing on NRC3.

The study shows how NRC1 and NRC3 proteins from various plant species exhibit different responses to SS15, with some variants being insensitive. They claim that this highlights evolutionary diversity within the NRC family in the Solanaceae species related to the interaction with SS15.

In previous studies, they examined the roles of positions D317 and E316 in NRC2. In this manuscript, they investigate how structural polymorphisms, particularly at positions E316 and D317 within the NB-ARC domain, are linked to the resistance of certain NRC proteins to SS15 suppression. The E316K substitution in NRC3 appears to be critical for modifying sensitivity across species, with lysine (K) at position 316 being associated with resistance to SS15.The authors show that polymorphism at NRC3 position 316 determines the ability of SS15 to suppress NCR3 function.

This provides a clear example of how a single amino acid can dictate protein-protein interactions. Throughout the evolution of NRC3, only two amino acids, K or E, have been present at this position, suggesting that this site was likely not under selective pressure to inhibit SS15 binding during evolution

I find this manuscript very interesting. Finding that position 316 is important to the function of the NRC protein can enhance our understanding of its function.

We are grateful for your positive comments on our manuscript and the thoughtful suggestions provided.

Weaknesses:

While the study focuses on the evolutionary dynamics of NRC proteins and their interaction with SS15, it doesn’t explore how these findings might influence crop breeding strategies to enhance disease resistance in agriculture.

Our study focuses on understanding the evolutionary dynamics of immune receptors against their suppressors, rather than directly addressing crop disease resistance. However, we recognize the potential agricultural applications of our findings. To bridge the gap between our phytopathological discoveries and real-world agriculture, we have established a startup company called *R*esurrect Bio (<https://resurrect.bio>). We aim to leverage these findings to enhance disease resistance in future projects.

The role of other residues beyond E316K is briefly touched upon (e.g., K314 in SlNRC3), but further exploration of these polymorphisms could strengthen the understanding of SS15 resistance. Their contribution to the insensitivity of tomato NRC3 could be elaborated.

Since our analysis utilized ancestral sequence reconstruction for the entire NRC3 interface against SS15, it comprehensively accounted for residues beyond E316K. This approach enabled us to identify the primary role of E316K in SS15 suppression. Furthermore, other residues within the interface that may contribute to SS15 evasion were also discussed in the paper.

The conclusions are based on transient expression assays, and it would be beneficial to discuss how these results might differ in stable transformation systems.

We acknowledge that the conclusions are drawn from transient expression assays. However, due to the diversity of NRC3 sequences from different species (*N. benthamiana*, pepper, tomato, and potato), it would be highly challenging to confirm these results in stable transformants for each species.

The manuscript is challenging to follow due to the excessive use of abbreviations. For example, what is the difference between NRC3 and NRC3^EEE^. The label in Figures 4, 6 S4-8, is confusing. Instead of specifying which antibody was used, it would be clearer to label the detected proteins directly.

In response to the feedback, we have clarified the meaning of "EEE" in the figure legends related to the BN-PAGE and SDS-PAGE assays to ensure that this abbreviation is clearly understood. However, we prefer to indicate the antibodies used rather than protein names because each antibody has specific detection properties.

Fig 3c-. Please elaborate on the experiment, the samples, the counts, and how you defined the scale of HR scores ("Cell death and suppression assays"- method section is unclear about the HR scores between 1-6).

The detailed experimental procedure for the cell death assays is described in Derevnina et al., 2021, and Segretin et al., 2014, both of which are cited in our manuscript. The cloning details are provided in S2 Table. Additionally, we have clearly indicated in the revised manuscript that all HR scores underlying the figures are available in S1 Data. To further clarify, we have added a supplementary figure illustrating the HR scoring scale (S13 Fig).

Evolutionary trade-offs of E-K transition- is it associated with other aspects of plant fitness?

We have discussed possible trade-offs between the E316 and K316 variants in the discussion section (lines 466–478). These hypotheses explore the potential impact of these polymorphisms on plant fitness beyond their interaction with SS15.
